# Corneal Buckling during Applanation and Its Effect on the Air Pressure Curve in Ocular Response Analyzer

**DOI:** 10.3390/ijerph16152742

**Published:** 2019-07-31

**Authors:** Agnieszka Jóźwik, Henryk Kasprzak, Agata Kozakiewicz

**Affiliations:** Department of Optics and Photonics, Wroclaw University of Science and Technology, Wybrzeze Wyspianskiego 27, 50-370 Wroclaw, Poland

**Keywords:** corneal buckling, Ocular Response Analyzer, air pressure curve

## Abstract

The paper presents, for the first time, corneal buckling, during the air puff applanation, recorded with use of Ocular Response Analyzer (ORA), when the cornea is deeper deformed after its applanation. Precise numerical analysis of the air pressure curve from the raw data, distinct local disturbances of the curve, which appear almost exactly at the time of the first and the second applanations. Thirty measurements taken on six eyes show clear dependencies between times of both applanations and appearances of local wave disturbances on the air pressure curve as well as between the amplitude of pressure wave disturbances and the respective height of applanation curve. These findings can be interpreted as a result of very fast corneal buckling, that produces the air pressure wave, propagating from the cornea towards the device. The quantitative dependencies measured and described in this study, enable to characterize the individual buckling during respective applanations. Due to these individual characterizations and dependencies it is possible to understand and describe better the ultrafast corneal applanation process. Such phenomena could likely be employed to increase the accuracy of measured parameters by ORA or for identifying new types of biomechanical properties of the cornea.

## 1. Introduction

Biomechanical behavior of the cornea is a factor affecting the results of various eye measurements and can be a source of important information, helping understand its biomechanical properties as well as to introduce new types of examination procedures and innovative ways to analyze properties of the cornea.

Ocular Response Analyzer (ORA, Reichert company) was the first device capable of estimating some biomechanical properties of the cornea in vivo [1,2]. Examinations performed with use of ORA are based on numerical analysis of two curves given by the device during measurement within 25 ms. The first curve describes the air pressure distribution exerting from the air jet of the device on the cornea, and the second one, called the applanation curve, corresponds to the intensity distribution of light, reflected from the corneal apex and captured by the built-in detector. The corneal applanations are manifested on the applanation curve in a form of distinct local maxima, where the intensity of light reflected from the corneal apex is the most concentrated.

A two-way corneal applanation process is used to calculate the intraocular pressure (IOPg) and the corneal compensated intraocular pressure (IOPcc). Moreover, ORA enables quantifying the biomechanical properties such as corneal hysteresis (CH) and corneal resistance factor (CRF). CH is described as corneal viscoelasticity, and according to Robert’s paper [3] CRF is a viscoelastic parameter, weighted by the Central Corneal Thickness (CCT) and highly correlated with CH.

**Figure 1:**
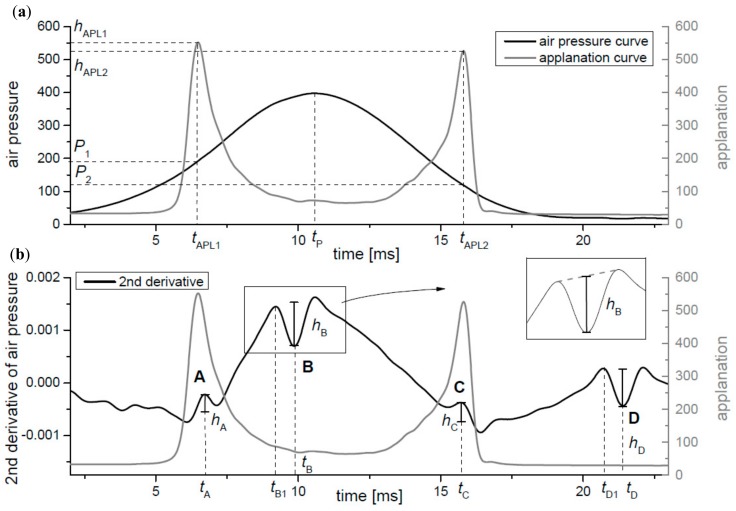
The applanation and the air pressure curves (**a**) and the second derivative of the air pressure curve (**b**) with parameters estimated from the curve. See text for model notation details and description of symbols.

Temporal fluctuations in CH and CRF values may contain information about changes in the cornea with regard to ocular pathologies, e.g., keratoconus, corneal collagen cross-linking, or glaucoma [4,5,6,7,8,9]. These basic four parameters given by the device, are calculated by the ORA software from the coordinates of two characteristic points determining respective pressures *P*_1_ and *P*_2_ on the air pressure curve, corresponding to two local maxima of the applanation curve during both applanation moments *t*_APL1_ and *t*_APL2_ (see Figure 1a).

ORA gives the possibility to export 400 raw data points for every curve, four basic parameters, and, additionally, 37 more parameters, depending on the form and quality of the applanation curve. As is stated in the literature [10,11], ORA’s air pump is switched off immediately after the first applanation, when the air pulse flattens the corneal surface in the apical region. However, the air pressure is still increasing and reaches its maximum after a few milliseconds from the first applanation. Simultaneously, the central cornea still keeps sinking up to the moment of maximal concavity. Next, the air pressure from the pump decreases and the cornea returns to the physiological shape passing the second, outward applanation.

The results of measurements show that the air pressure curves can differ significantly for a particular subject, which means that the pump is switched off at different applanation times, *t*_APL1_. Some authors describe the characteristics of air pressure curve as symmetrical [10] or having the shape of a Gaussian distribution [11], whereas our previous study [12] showed that this curve does not seem to be symmetrical, but can be characterized by a slower “increase” and a faster “decrease” in air pressure over time. One of the explanations may be associated with the viscoelastic properties of air, which could affect the measurement results. Additionally, the measurements performed on a rigid glass lenses [12] presented the lack of applanation associated with almost identical air pressure curves for every single measurement, due to complete pressure ejection from the pump. Access to raw data offered by ORA device, gives possibility to define own, new parameters of the anterior eye, which can be useful in new approach to ophthalmic diagnosis [13].

In the contrary to the applanation curves, more detailed analysis of the form of the air pressure curve provided by ORA instrument has not been carried out so far. Fine numerical analysis of the air pressure curve shows existing four local waveform disturbances of the curve. The most surprising result is that two out of the four small, local wave disturbances in the air pressure curve are likely correlated with the both peaks of applanation curve. The source of recording of both curves in the device is totally different, so the reason of observed effect seems to be quite an important and interesting phenomenon. We claim that this effect is a result of corneal buckling, which is an instability that occurs during both applanations. The concept of buckling in eye research is usually understood as scleral buckling which is a surgical procedure used in ophthalmology to treat retinal detachments [14] and is often presented in literature.

The buckling effect during corneal applanation was already mentioned in a few papers. Marg et al. [15] proposed new tonometer based on a contact tonometry examination, in which the applanation is achieved mechanically. They observed two local crests on the response curve of their new tonometer, and tried to explain it by use of bending or buckling effect. However, they finally reported that according to their analysis “corneal rigidity rather than buckling is responsible for the crest…” The modeling of corneal applanation during Goldmann tonometry measurement by use of Finite Element Method (FEM) by Srodka [16] introduces buckling to explain the effect of a much larger magnitude of corneal displacement after applanation than before, using the same force. Widlicka-Asejczyk et al. [17] suggested that applanation of the corneal shell loaded with a high IOP is accompanying by buckling. Additionally, they claim directly that taking into account the corneal apex buckling during applanation gives the closest correction of Goldmann tonometry IOPg to IOP value, obtained with dynamic contour tonometry.

The compressive stress of the cornea during mechanically-induced applanation causes buckling, which is manifested as a sudden and extremely quick redistribution of forces inside the cornea. Corneal deformations during mechanically-induced applanation are much slower than in case of the air puff forced deformations of the corneal apex. In the case of air puff tonometers, the redistribution of internal stresses in the corneal apex during its applanation occurs within fraction of one millisecond. Such an extremely fast mechanical effect inside the cornea flutters its apex during applanation, and can generate the ultrafast air pressure pulse of the form of wave. It is likely that this air pressure pulse from the corneal apex overlaps the air puff from the tonometer and is also captured by the manometer of the device, finally causing some fine, local disturbances of the air pressure curve.

The aim of the work presented here is to quantitatively analyze the occurrence of the four characteristic disturbances in the air pressure curve, observed during tonometry measurements taken with the use of ORA. Such analysis could give a new look at biomechanical behavior of the cornea during its deformation. Two of these local disturbances are clearly associated with both applanations. Possible source of two others is discussed in the last section. Geometry and time analyses of the wave disturbances of the air pressure curve show their clear correlations with separately, but synchronously recorded applanation curve. Such similarities and correlations presented in the paper may confirm our hypothesis on corneal buckling during the corneal applanation and its influence on the applanation curve.

## 2. Materials and Methods

The raw data of the applanation and the air pressure curves were obtained directly from the ORA device (Reichert Ophthalmic Instruments, Depew, NY, USA, the model from the year 2009). An exemplary shape of both curves is shown in the Figure 1a. Units shown on the left and the right vertical axis presents units of raw data given by the ORA device both for the air pressure and the applanation values. All following calculations were based on these units and marked as (a.u.). Both curves were smoothed numerically with the Gaussian filter with the size window allowing to remove a local noise of curves, without losing information characterizing all observed small wave disturbances.

The obtained curves were interpolated by use of cubic splines in order to increase number of data points from 400 to 4000. The time of the first (*t*_APL1_) and the second (*t*_APL2_) applanations and their corresponding maxima (*h*_APL1_ and *h*_APL2_) were calculated from the applanation curve. The time for the maximum value of the air pressure *t*_P_ was calculated from the smoothed air pressure curve. Estimated parameters are presented in the Figure 1a. The first and the second derivatives of the air pressure curve were calculated numerically in order to find its local disturbances. Four characteristic local wave disturbances, marked as A, B, C, and D, can be clearly seen in the plot of the second derivative (Figure 1b) that has especially proven to be sensitive to visualize the local disturbances of the air pressure curve. Next, times of appearance of characteristic extreme points of disturbances (*t*_A_, *t*_B_, *t*_C_, and *t*_D_) were estimated. When one compares the second derivative of the air pressure curve to the applanation curve, it can be clearly seen that two of these characteristic disturbances (A and C) correspond to applanation times (*t*_APL1_ and *t*_APL2_). To better visualization of this, the values of the second derivative curve have been reversed to maintain the similar shape of the disruption A and C to the applanation peaks (they were both local maximum values) (Figure 1b). The other two disruptions (B, D) occur after the first and the second applanations. The respective heights of all local disruptions were determined (*h*_A_, *h*_B_, *h*_C_, and *h*_D_). Due to the lack of symmetry of all four local irregularities, their heights were calculated in relation to line connecting both neighboring local extreme points, as it is outlined in Figure 1b. Additionally, times *t*_B1_ and *t*_D1_ on the air pressure curve were also calculated, when disturbances B and D begin, respectively.

In order to investigate the parameters in real-life application, preliminary screening trials were performed. Six volunteers without any eye disease took part in the study. The group with the similar age (25–31 years) was taken for examination to eliminate age-dependent factors. Only one eye of each person was measured. Measurements were repeated 30 times. Participants were informed about the process of the noninvasive methods of measurement. The project was approved by the Ethics Committee of Wroclaw Medical University (KB 481/2009) and complies with the Tenets of the Declaration of Helsinki.

## 3. Results

**Figure 2:**
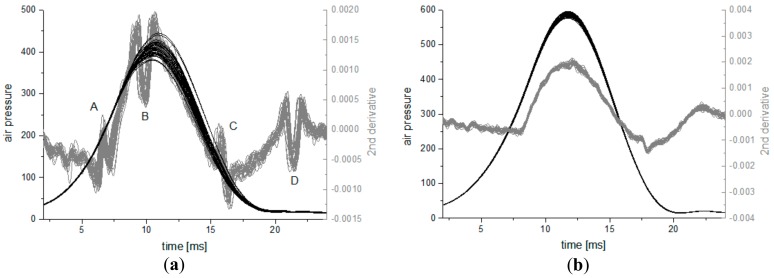
(**a**) Air pressure curves and their second derivative for 30 measurements on one participant (subject no. 1) and (**b**) 30 air pressure curves and their second derivatives for the rigid glass lens.

The repeatability localization and shape of disturbances observed by means of the second derivative of the air pressure curve estimation precludes their complete randomness. The local disturbances A, B, C, and D are marked in the Figure 2a. Analogous measurements with the same device were carried out on plano-convex rigid glass lenses (BK7) with radius of curvature equal to 8.9 ± 0.1 mm [12]. Reproducibility of 30 air pressure curves and their second derivatives measured on glass lens is much higher (Figure 2b) than these observed for the subject’s eye. There are some small local oscillations on the second derivative measured on the glass lenses, especially at the moment of about 18 ms from the beginning. Measurements on another two glass lenses with curvature radii of 8.7 and 9.0 mm show a very similar behavior in shape of curves as in the Figure 2b, with similar oscillations, located in the same places.

**Figure 3:**
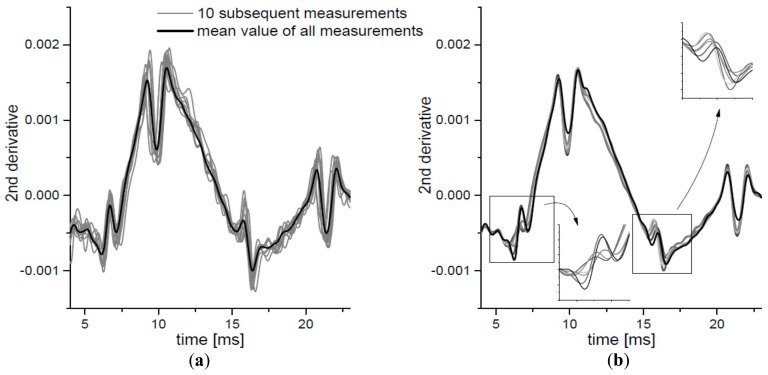
(**a**) The second derivative of the pressure curve for 10 subsequent measurements for one participant (gray lines). The black line is the mean value of 30 measurements for this participant. (**b**) The mean value for 6 participants. See text for detailed description of the insets.

The mean values of the second derivatives of the air pressure curve for all 30 measurements taken for one subject were calculated. The result is presented as the black curve in the Figure 3a with the air pressure curves obtained for each single measurement. For the clarity of the plot, only series of the first 10 curves for one subject is shown (gray lines) in the figure. Six curves calculated as the averaged second derivatives of air pressures from measurements captured for six subjects are presented in the Figure 3b. Disturbances A and C, related with both applanations, appear in slightly different times for each person and also they clearly differ in their heights. Different observation is in the case of averaged disturbances B and D, which occur in the same time independently from the examined person.

**Figure 4:**
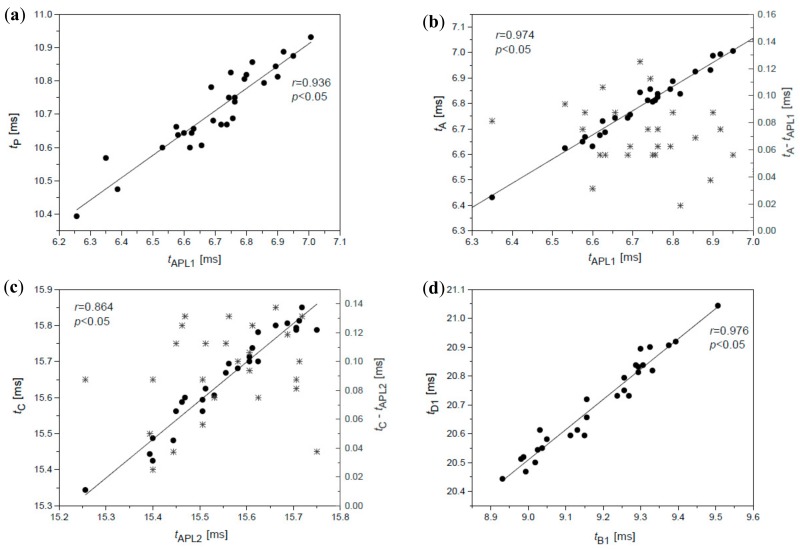
Dependence between the time of the first applanation *t*_APL1_ and the time of maximum pressure *t*_P_ (**a**), the time of the first applanation t_APL1_ (dots) or the difference between time *t*_A_ and *t*_APL1_ (stars) and the time of first disruption *t*_A_ and vs. (**b**), and the time of the second applanation *t*_APL2_ (dots) or the difference between time *t*_C_ and *t*_APL2_ (stars) and the time of the third disruption *t*_C_ (**c**), time of disturbance *t*_B1_, and time of disturbance *t*_D1_ (**d**) for participant no. 2.

Dependence between time of the first applanation *t*_APL1_ and time of the maximal value of the air pressure *t*_P_ (Figure 4a and Table 1) is strong, but these correlations are expected since the air pump of the device is switched off just after the first corneal applanation, after the time *t*_APL1_.

An interesting and rather surprising finding is the occurrence of local disturbances A and C of the air pressure curve, which is clearly related to both applanations observed in all measurements. Relations between the time of the first applanation *t*_APL1_ and the respective time *t*_A_ of the disturbance A (Figure 4b) as well as between time of the second applanation *t*_APL2_ and time *t*_C_ of the disturbance C (Figure 4c) are direct and surprisingly very high. Values of Pearson correlation coefficients for these dependencies are presented in Table 1. The respective values of differences *t*_APL1_–*t*_A_ and *t*_APL2_–*t*_C_, given on the right axes of both figures (Figure 4b,c), show time interval after which the disturbance with respect to applanation occurs. The average value of the first difference is smaller and amount to about 80 μs while the average value of the second difference is ~120 μs for this subject.

The open question concerns origin of disturbances B and D on the second derivative of the air pressure curve, which are not related to appearance times of A and C oscillations nor with the respective occurrence of both applanations (*r* < 0.2). Numerical analysis showed that disturbances B and D are dependent on each other. Times *t*_B_ and *t*_D_ of characteristic extremes of both oscillations are strongly correlated, while correlations between times *t*_B1_ and *t*_D1_ are even stronger (Figure 4d; Table 1), with Pearson correlation coefficient higher than 0.9. The heights of disturbances B and D are also highly correlated.

**Figure 5:**
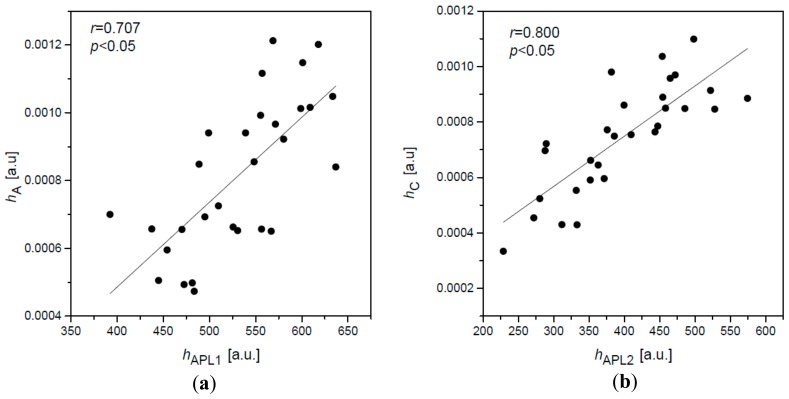
Dependence between the height *h*_A_ of the A oscillations of the 2nd derivative and the height *h*_APL1_ of the first applanation peak (**a**), the height *h*_C_ of the third oscillation C, and the peak height *h*_APL2_ of the second applanation (**b**) for the subject No. 2. Units of both axes refer to air pressure units (second derivative) and the applanation curve units, respectively.

Analysis of heights of disturbances A and C manifests even more unexpected results. Heights of two peaks *h*_APL1_ and *h*_APL2_ of applanation curve are correlated with heights (amplitudes) *h*_A_ and *h*_C_ of disturbances A and C (Figure 5 and Table 1). Higher correlations of respective heights are observed for the second, outward corneal applanation (*h*_APL2_ and *h*_C_) in comparison to the first one (*h*_APL1_ and *h*_A_) for all six subjects. This is likely not accidental and have some particular reason.

**Figure 6:**
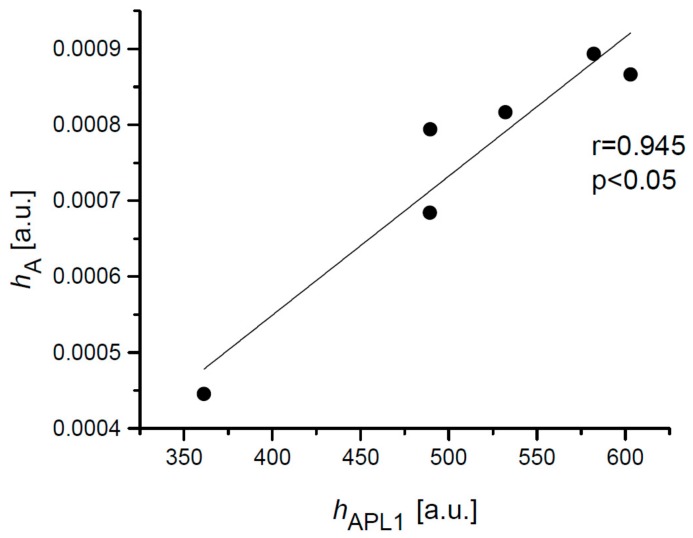
Correlation between the mean height *h*_A_ of the A oscillations of the second derivative and the mean height *h*_APL1_ for 6 subjects.

The values of the heights *h*_A_, *h*_C_, *h*_APL1_, and *h*_APL2_ were averaged for each subject and correlations between *h*_A_ and *h*_APL1_ with the correlation coefficient 0.945 (*p* < 0.05) was estimated (Figure 6). Similar correlation was calculated for the height *h*_C_ and height *h*_APL2_ with *r* = 0.899 (*p* < 0.05).

It has been noticed that the local peak of oscillation A usually occurs shortly after the first applanation, while the temporal difference between the highest peaks of oscillation C in relation to appearing of the second applanation is greater. In order to verify this observation, the *TR* parameter (time relation) was used as the quotient of differences between the times of the disturbances with respect to the corresponding applanation times:

(1) $$TR=\frac{t_{C}\;-\;t_{\mathrm{APL}2}}{t_{A}\;-\;t_{\mathrm{APL}1}}$$

**Figure 7:**
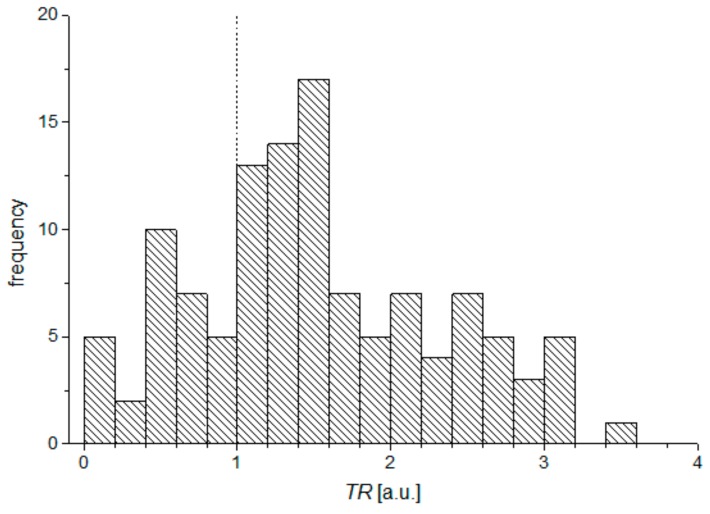
Histogram of *TR* parameters for all measurements.

Histogram of *TR* parameter for all measurements is presented in the Figure 7.

Higher differences between *t*_C_ and *t*_APL2_ than between *t*_A_ and *t*_APL1_ are observed for 75% results.

## 4. Discussion

The results of fine numerical analyses of the air pressure and applanation curves recorded in 30 measurements on six examined eyes, presented above, show some unexpected findings. The air pressure curve represents the pressure distribution of the air puff from the device pump. However, the smooth-looking air pressure curve contains four, insignificant at a glance, local wave disturbances (A–D), which are not visible at the first sight. The second derivative of the curve turned out to be sensitive enough to visualize these disturbances.

Times *t*_A_ and *t*_C_ of the appearance of the wave form disturbances A and C are clearly and highly correlated with times of two corneal applanations *t*_APL1_ and *t*_APL2_. The question is: How is it possible for both corneal applanations to influence the two local disturbances in the distribution of the air pressure from the pump?

It can be explained as a result of the corneal buckling during both applanations, which generates ultrafast, local disturbances of the air pressure, overlapping the air puff from the device pump. Analysis of respective differences between *t*_APL1_ and *t*_A_, and *t*_APL2_ and *t*_C_ show that disturbance A appears usually in shorter time after the first applanation than disturbance C after the second applanation. Average values of time differences amount to 70 μs and 100 μs, respectively. Additionally, taking into account Equation (1) and analyzing of the Figure 7 it can be concluded that in most cases, the difference between time *t*_C_ and *t*_APL2_ is greater than the difference between *t*_A_ and *t*_APL1_. There might be different possible explanations of this effect. One of them is that this it is likely associated to the biomechanical properties of the cornea during applanation. The relation between respective heights *h*_A_ and *h*_C_, describing disturbances A and C on the second derivative of the air pressure curve, and the respective heights of applanation peaks *h*_APL1_ and *h*_APL2_ of the applanation curve are also very strong, and is, again, a surprising finding. The values of respective correlation coefficients, given in the last two columns of the Table 1, show relatively high dependencies between considered heights for all examined subjects. The higher magnitudes of peaks mean the more the concentrated light beam reflected from the oblate corneal apex reaches the built-in photodetector of the instrument, which is related to smoother corneal flatness during its applanation or a wider area of corneal flattening. Such a form of the applanated corneal apex can be characterized as “a better applanation” of the cornea, which affects the magnitude of appropriate disturbances A and C (Figure 5). It can be explained that “the better corneal applanation” is accompanied by stronger buckling, which produces more energy, and thus the amplitude of the air pressure disturbance is higher. Thus, the better corneal applanation is somehow related with the stronger buckling effect. This property of “buckling quality” of individual cornea during applanation needs deeper and more sophisticated extended analysis. Moreover, the ideal applanation effect is relatively idealistic, due to the influence of the tear film quality or high frequency of the corneal vibrations during applanations on the quality of reflected signal. A real study of the maximum reflection during both applanations can make future research more realistic and closer to clinical practice. Viscoelasticity of the corneal structure may also have some impact on measured differences between discussed correlations during inward and outward applanations and occurring buckling. High correlation between heights *h*_A_ and heights of applanation peaks *h*_APL1_ is manifested not only in the results of several measurements for one particular subject, but also six heights *h*_A_ and *h*_APL1_ averaged from all measurements for every subject (Figure 6). It likely means that the individual cornea has the tendency to demonstrate greater applanation in relation to other corneas.

The explanation of the origin of wave disturbances B and D of the air pressure second derivative is more complex than the understanding of the origin of disturbances A and C. It seems certain that these two disturbances are not related to corneal applanations. Calculations showed a very low correlation between respective time’s *t*_APL1_ and *t*_B_ or *t*_APL2_ and *t*_D_ as well as between their respective heights (*r* < 0.2). However, high correlation coefficients between respective times of appearing for disturbances *t*_B_ and *t*_D_ as well as *t*_B1_ and *t*_D1_ (Figure 4d and Table 1) indicate relatively high dependency between both disturbances. Furthermore, the averaged values of times *t*_B_ and *t*_D_ for all six subjects are almost the same, unlike the respective times *t*_A_ and *t*_C_. We suppose that disturbances B and D do not originate from the cornea, but are related to the operation of the air pump. Perhaps disturbance B characterizes the time, when the air pump piston stops after the air ejection, while the disturbance D shows the effect when the piston returns to the initial position after the measurement.

Additionally, for the test purposes, the analysis presented in the study were performed on the results obtained with use for three different ORA devices for 10 measurements on one eye of two of coauthors of the paper. One ORA instrument from was the same model as the one used in the main study and two others were new generation instruments. The appearance of the same four disturbances on the second derivative of the air pressure curve was, again, clearly observed in results obtained for the same generation model. However, measurements performed with use of the newer models did not show the appearance of disturbances B and D, while disturbances A and C were observed, but they were only slightly weaker. It is very likely the different designs and ways to manage the air flow and air pressure measurement in the new generation ORA models reduced the magnitudes of all four disturbances.

## 5. Conclusions

The results presented in the paper show that very fast corneal buckling occurs during corneal applanation in air puff tonometer. This buckling generates an ultrashort air pressure pulse, which can be recorded out of the cornea. Taking into account the moments of appearances of this buckling may—in the future—improve determination of both applanations in the air puff tonometer and finally may increase the accuracy of IOP measurement. Additionally, the amplitude of the air pressure pulse caused by the corneal buckling may characterize the buckling itself and “quality” of both corneal applanations. More extended examinations of these effects and their application for investigation in optometric and ophthalmic practice may enable better understanding of the corneal properties and their differentiations for specific eye pathologies. It would be interesting and valuable to explore the effects with abnormal eyes.

## Figures and Tables

**Table 1:** Pearson correlation coefficients for different dependencies for all participants.

Subject No.	*t*_P_ vs. *t*_APL1_	*t*_A_ vs. *t*_APL1_	*t*_C_ vs. *t*_APL2_	*t*_B_ vs. *t*_D_	*t*_B1_ vs. *t*_D1_	*h*_B_ vs. *h*_D_	*h*_A_ vs. *h*_APL1_	*h*_C_ vs. *h*_APL2_
1	0.902	0.973	0.871	0.818	0.957	0.874	0.622	0.803
2	0.936	0.974	0.971	0.756	0.976	0.849	0.707	0.800
3	0.762	0.808	0.917	0.720	0.912	0.822	0.843	0.874
4	0.648	0.951	0.836	0.886	0.967	0.857	0.756	0.853
5	0.649	0.950	0.970	0.961	0.993	0.692	0.813	0.845
6	0.850	0.991	0.938	0.952	0.992	0.942	0.703	0.829
